# Applications of Chemical Shift Imaging to Marine Sciences

**DOI:** 10.3390/md8082369

**Published:** 2010-08-19

**Authors:** Haakil Lee, Andrey Tikunov, Michael K. Stoskopf, Jeffrey M. Macdonald

**Affiliations:** 1 Joint Department of Biomedical Engineering NC State University and UNC Chapel Hill, Chapel Hill, NC 27599, USA; 2 Environmental Medicine Consortium, NC State University, 4700 Hillsborough St., Raleigh, NC 27606 USA; E-Mails: tikunov@gmail.com (A.T.); michael_stoskopf@ncsu.edu (M.K.S.); jeffrey_macdonald@med.unc.edu (J.M.M.); 3 Department of Clinical Sciences, College of Veterinary Medicine, North Carolina State University, 4700 Hillsborough St., Raleigh, NC, 27606, USA

**Keywords:** oyster, Crassostrea virginica, magnetic resonance imaging, chemical shift imaging, carbon, glycine, betaine

## Abstract

The successful applications of magnetic resonance imaging (MRI) in medicine are mostly due to the non-invasive and non-destructive nature of MRI techniques. Longitudinal studies of humans and animals are easily accomplished, taking advantage of the fact that MRI does not use harmful radiation that would be needed for plain film radiographic, computerized tomography (CT) or positron emission (PET) scans. Routine anatomic and functional studies using the strong signal from the most abundant magnetic nucleus, the proton, can also provide metabolic information when combined with *in vivo* magnetic resonance spectroscopy (MRS). MRS can be performed using either protons or hetero-nuclei (meaning any magnetic nuclei other than protons or ^1^H) including carbon (^13^C) or phosphorus (^31^P). *In vivo* MR spectra can be obtained from single region of interest (ROI or voxel) or multiple ROIs simultaneously using the technique typically called chemical shift imaging (CSI). Here we report applications of CSI to marine samples and describe a technique to study *in vivo* glycine metabolism in oysters using ^13^C MRS 12 h after immersion in a sea water chamber dosed with [2-^13^C]-glycine. This is the first report of ^13^C CSI in a marine organism.

## 1. Introduction

Marine and estuarine ecosystems are valuable, biodiverse resources threatened by climate change and other environmental insults [1,2]. In some instances, marine life has shown the ability to adapt to extreme environmental conditions and in others there are failures to adapt to changing conditions and anthropogenic insults [3]. It is imperative that we understand how marine life responds to its environment and to environmental stressors, both genetically [4] and metabolically. To date, much of the anatomic, physiologic and biochemical data on marine organisms has been gleaned from studies of *ex vivo* tissue samples [5]. This limits the ability to do longitudinal studies, impedes the study of rare protected species, and most importantly, precludes dynamic stress-response studies in the intact, living organism. Magnetic resonance imaging (MRI) and spectroscopy (MRS) are proven tools that overcome these limitations. *In vivo* applications of MRI and MRS have evolved and matured into numerous diversely-defined fields and excellent reviews are available [6–9]. Along with the development of numerous imaging applications, steady improvement of magnet hardware has provided instruments with higher magnetic field strengths than the typical 1.5 Tesla (T) or 3 T magnets found in routine clinical settings, enabling improved fundamental research on physiological phenomena [10]. Application of these magnets and advanced MR methods to marine systems, however, must address unique challenges due to the physical characteristics of seawater and the complex life support needs of marine animals, as detailed in a seminal paper addressing these problems [11]. Overcoming these technical challenges has resulted in numerous reports of the use of MRI and MRS in marine science, including both anatomic and functional studies such as the examination of flow and oxygen concentrations in specimens subjected to various stressors [12–14]. More recent studies include the examination of a variety of marine animals including scallop, oyster, mussel, cod, crab and sea urchins [5,15–17]. The need for further work using invasive techniques to establish the foundation for interpretation of non-invasive studies remains, but the application of advanced imaging techniques is now a real possibility in marine science investigations.

MRI is based on the nuclear magnetic resonance (NMR), a well known analytical method of chemistry and molecular structural biology. MRI is accomplished by supplementing basic NMR analytical equipment with gradient coils, a hardware component that shifts the frequencies emitted by relaxing nuclei in the tissues of a subject in a manner consistent with their spatial coordinates along different axes. This makes it possible to generate images by deciphering the emitted frequencies according to the spatial locations of nuclei and translating the concentration of nuclei contributing to each frequency to proportional image intensities for each unit of the image [7,8]. One particularly attractive feature of MRI techniques is that they do not use any ionizing radiation, but do permit visualization of the interior of subjects. This lack of ionizing radiation makes it possible for an object to be scanned repeatedly without known side effects. The closely related techniques of *in vivo* MRS provide spectral information on the small mobile metabolites participating in various metabolic pathways from user defined regions inside an organ, tissue or material. MRS spectroscopy examines the modulation of radio frequency emissions of relaxing magnetic nuclei in those molecules and is capable of identifying the presence of a range of pathological conditions in a sample or specimen [18,19]. MRS is based on the chemical shifts that occur when the resonance frequency of a nucleus is modified by its surrounding chemical environment. By measuring the distribution of chemical shifts from a sample it is possible to identify a particular molecule. Most commonly, *in vivo* MRS is performed on the most abundant magnetic nucleus, the proton (^1^H). The abundance of the proton allows scanning to overcome the issue of signal to noise constraints relatively rapidly and permits data acquisition in a relatively short time. Many important metabolites can be easily measured by examining proton spectra. These include *N*-acetyl aspartate, choline and creatine. MRS data can be acquired either from a single volume of interest (VOI) or region of interest (ROI), which is also often referred to as a voxel. It can also be acquired from multiple voxels by the techniques commonly referred to as either chemical shift imaging (CSI) or synonymously MR spectroscopic imaging (MRSI) [20]. Single voxel MRS provides the spectrum from a selected cube-shaped ROI, whose dimension and location can be controlled precisely by the user [21,22]. CSI is a technique based on imaging sequences, but with the modification that during data acquisition the read out gradient is omitted to conserve chemical shift information. From this multi-dimensional spectral data array, the chemical shift can be visualized from multiple small voxels located within a large examined area, and the registered signal forms a map of spatial distribution of the metabolites being studied [23,24].

In this paper we report the application of CSI to marine samples along with a brief description of the technique. We also provide evidence of *in vivo* glycine metabolism in the Eastern oyster (*Crassostrea virginica*) using ^13^C MRS and CSI acquired 12 hrs after exposure to sea water dosed with [2-^13^C]-glycine. This is the first report of ^13^C CSI of a marine organism.

## 2. Results and Discussion

**Figure 1:**
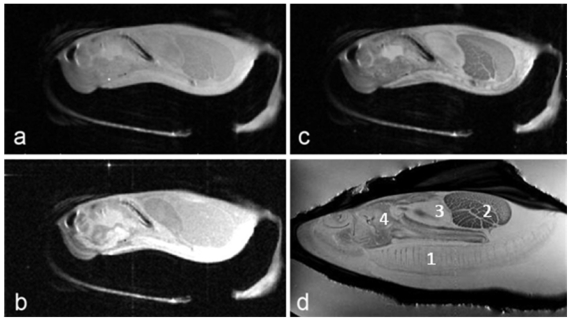
(**a**) Proton density (**b**) T1- and (**c**) T2-weighted images of an Eastern oyster (*Crassostrea virginica*). (**d**) T2-weighted image of another oyster in a life support system. 1: gills, 2: adductor muscle and hemolymph sinuses, 3: heart, 4: stomach. Note the fine anatomical structure displayed in the image with the gills fully extended.

**Figure 2:**
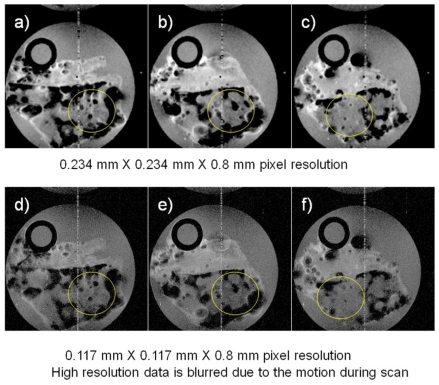
Three consecutive images chosen from a set of T_2_-weighted multi slice images of a marine sponge with a 0.8 mm slice thickness are displayed. The dark circle in the upper left corner of the image is the influent tube for sea water circulation by the peristaltic pump. Two different in-planar pixel resolutions were acquired [0.234 × 0.234 mm (images a, b and c) and 0.117 × 0.117 mm (images d, e and f)], showing that the higher pixel resolution image is not as sharp (for example, the area indicated within the yellow circles). This was expected and is due to the motion of the sample created by the pulsatile movement of the circulating sea water. The number of averages was two and the experimental times for upper and lower row images were 512 and 1,024 seconds, respectively.

**Figure 3:**
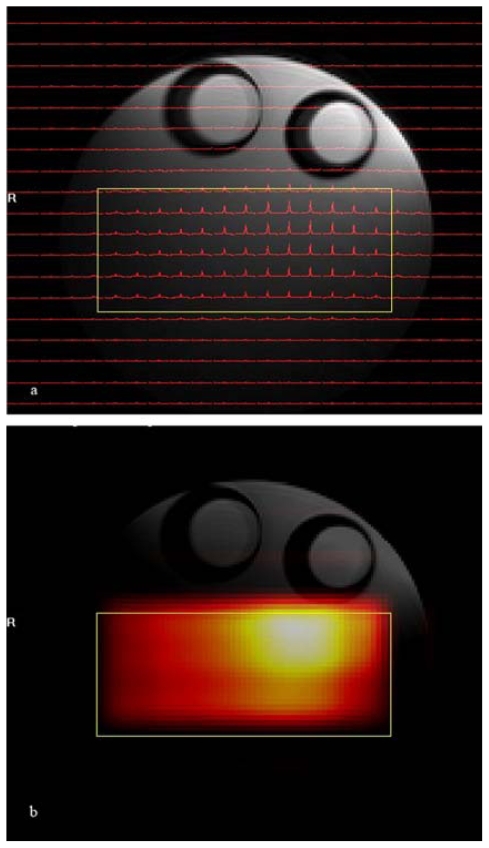
CSI was performed after selecting the volume of interest (yellow rectangle) by PRESS sequence. The CSI data were displayed as either a small array of spectra aligned according to their spatial origins after being extrapolated to 32 × 32 from the original 16 × 16 data matrix size (**a**) or as a metabolic map image whose pixel intensity is proportional to the area under the peak after peak integration between predefined spectral ranges. In this case the peak shown in (**b**) has been extrapolated to 128 by 128. The images were enlarged for better viewing.

**Figure 4:**
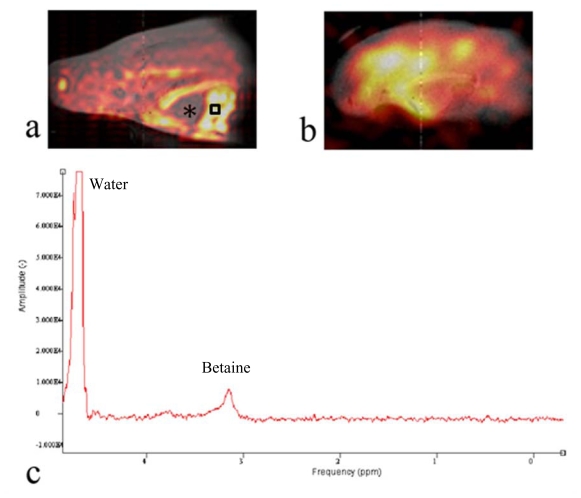
A metabolic map from an Eastern oyster 12 h after exposure to [2-^13^C]-glycine inside a sea water chamber was obtained by using ^13^C CSI. The ^13^C metabolic map enables one to detect the spatial distribution of metabolites, revealing the distribution of betaine (**a**) including a low concentration in the heart marked with asterisk, and glycine (**b**). A proton PRESS sequence was used to obtain the localized spectrum within the abductor muscle of the oyster using a voxel size of 2 mm × 2 mm × 2 mm (square). The spectrum shows the remaining water peak (*δ* 4.7) from incomplete water suppression and betaine (*δ* 3.2)(**c**).

Image contrast in MRI depends on many different properties of tissue. T1 and T2 are tissue state specific time constants that are related to how quickly the nuclei will emit their absorbed radio frequency energy (RF, 200 MHz for our case when using a 4.7 Tesla magnetic field strength) and lose coherence through molecular interactions, respectively. The image contrast is also dependent on the concentration of protons in the sample tissue. Controlling the scan parameters or timings can maximize the effects of T1, T2 or proton density and the resultant images are typically called T1-, T2- or proton density weighted images to denote the main parameter determining image contrast. Many other parameters including macroscopic and microscopic motions of proton nuclei can also be utilized as parameters to determine image contrast. For example, macroscopic motions can be used in MR angiography and microscopic motions can be used to obtain diffusion weighted images. These different scan parameters are called MR pulse sequences, and acronyms are commonly used to identify specific applications of these MR pulse sequences [7,8]. Here we present examples of both MRI (Figures 1 and 2) and MRS (Figures 3 and 4) with potential applications in the marine sciences.

Proton-density, T1- and T2- weighted images of the Eastern oyster (*Crassostrea virginica)* are shown in Figure 1. The long axis of the oyster was aligned along the main axis of the magnet and the short axis of the oyster was aligned along the horizontal diameter of the magnet bore. The slice orientation was parallel to the horizontal diameter of the magnet bore, an orientation that is commonly called a coronal section in clinical radiological terminology.

The images in Figure 1 of the oyster outside of a life support system and directly inside an 8 cm volume coil without a sea water jacket (Figures 1a–1c) were collected using a multi-slice spin echo sequence with TR/TE (time of repetition/time of echo) settings of 2,000 ms/8 ms (proton-density), 300 ms/8 ms (T1-weighted), and 2,000 ms/30 ms (T2-weighted), respectively. The number of averages in each case was two. Therefore the image acquisition times were 512 (proton-density), 77 (T1) and 512 (T2) seconds. The field of view was 8 cm × 4 cm with 256 and 128 pixels acquired, producing 0.31 × 0.31 mm pixel resolution for each image. The slice thickness was 1 mm. Typically 13 to 15 slices were acquired using each technique and Figure 1 shows only one of those slices. For Figure 1d, the oyster was in a constantly flowing sea water life support system. Note the gills are fully extended with seawater flowing across them when submerged in flowing water, unlike the images in Figures 1a–c.

Other MRI experiments were performed examining marine sponges inside a polypropylene tube chamber fitted with tubes to permit sea water circulation using a peristaltic pump and these images are displayed in Figure 2. The field of view for the images shown in Figure 2 was 3 cm × 3 cm and the TR/TE was 2,000 ms/30 ms for both acquisitions, but the experimental time for the higher resolution image was twice that of the lower resolution image because of twice the number of phase encoding steps being required (128 *vs.* 256). We attribute the failure of the higher pixel resolution image to be sharper than the lower resolution images to motion of the specimen caused by the pulsatile motion of the circulating sea water. This occurred even though the timing of the start of the MRI sequence was synchronized to the pump movement. This demonstrates that the challenges of improving resolution in flowing sea water systems. Even though it is possible to improve the nominal pixel resolution with longer acquisition times, the actual resolution gain is not necessarily what one expects when considering the potential motion of the specimen during data acquisition.

The results of the phantom ^1^H CSI experiment are shown in Figure 3. In that figure spectra are shown for the central 22 by 19 voxels of the total of 32 × 32 voxel images. The volume of interest, selected by using a combination of point resolved spectroscopy and an outer volume suppression method, is centered inside the conical 26 mm diameter tube filled with isopropyl alcohol and is marked by a yellow rectangle in the figure. As explained earlier ^1^H CSI data can be displayed as either a small array of spectra aligned according to their spatial origins (Figure 3a) or as a metabolic map created after peak integration between predefined spectral ranges of observed metabolites (in this case, the methyl group of isopropyl alcohol) which are overlaid over the reference proton image (Figure 3b).

The uneven intensity variation of the metabolic map is the result of changes in receiver coil sensitivity over the volume of interest, which is characteristic of the loop coil used in this experiment. Combinations of outer volume suppression and PRESS (point resolved spectroscopy)[25] voxel selection provides excellent suppression of signals from the surrounding voxels of isopropyl alcohol solvent and corn oil. Because the ^1^H CSI technique can acquire spectra from the several different voxels simultaneously, the ^1^H CSI method is suitable for analyzing the regional anatomic and functional heterogeneity of metabolites [20,24,26].

Tracer methods using NMR observable stable isotopes such as ^13^C glucose or pyruvate offer excellent possibilities for tracking the conversion kinetics of metabolic precursor compounds and metabolic fluxes *in vivo* [27,28] because these compounds form the carbon backbone of many metabolic precursors. It is therefore possible to label compounds along their metabolic pathways. Measuring metabolic fluxes in this way represents a powerful method to elucidate bioconversion pathways both qualitatively and quantitatively to provide insight into specific metabolic pathways involved in the metabolism of compounds of interest. NMR can detect the position of the isotopic labels in a molecule through chemical shift differences without chemical derivatization or destruction of samples.

To detect and determine the spatial distribution of ^13^C labeled metabolic products of the oyster, we used a ^13^C CSI method and the results are displayed in Figure 4. Similar to Figure 3, the ^13^C CSI results were overlaid on ^1^H reference images. Peaks around betaine and glycine were integrated separately and the integral values determined the pixel intensities shown in Figures 4a and b. This revealed the spatial distribution of ^13^C betaine (Figure 4a) and [2-^13^C]-glycine (Figure 4b) after the oyster was injected with 0.1 cc of 100 mM [2-^13^C]-glycine (Cambridge Isotope, Andover, MA, USA) into the digestive gland, and maintained overnight (ca 12 h) out of water. At that time, a 2 h acquisition of a ^13^C CSI was acquired and MR images of glycine and betaine were generated.

Figure 4b shows that glycine is not distributed evenly throughout the oyster. The signal appears to dissipate away from the point of injection. Perhaps if -^13^C CSI images were obtained sequentially after injection one could obtain whole distribution kinetics of the injected compound. However, a metabolite of glycine is the osmolyte, betaine. The image in Figure 4a shows that betaine is distributed across oyster’s body especially in cellularly dense tissues (muscle, part of mantle and intestine). The oyster heart is filled with hemolymph of very low cellularity and appears in the image as an area of low intensity pixels indicating low levels of betaine (Figure 4a, marked with “*”). This is primarily because of the low density of cells and likely low betaine levels in the hemolymph, but flow effects of the hemolymph could also dampen the signal. Most important is that the betaine distribution in the oyster (Figure 4a) does not reflect the distribution of glycine (Figure 4b). Seeing this interesting pattern, we performed high resolution NMR spectroscopy of tissue extracts from various organ blocks dissected from oysters to determine the degree of ^13^C-labeling of betaine under the conditions used for the imaging studies. Our extraction study showed that ^13^C-NMR spectra of *Crassostrea virginica* are dominated by natural abundance peaks of the osmolytes betaine (*δ* 56.4 and 69.3 ppm) and taurine (*δ* 38.3 and 50.5)[29]

To determine the metabolic profile around the area of high ^13^C signal in Figure 4a, we obtained a localized ^1^H-NMR spectrum from the oyster muscle. The PRESS sequence was used to obtain a ^1^H-NMR spectrum from a 2 mm × 2 mm × 2 mm voxel inside the aductor muscle (black square) after chemical shift selective pulses with dephasing gradients were applied to suppress water. The TR/TE was 1500 ms/15 ms and the number of excitations was 32. Figure 4c shows the ^1^H-NMR spectrum from the muscle in the oyster with a prominent betaine peak at *δ* 3.25 representing the trimethyl amine moiety. The chemical identity was confirmed by the high resolution NMR experiments after extraction [29]. This preliminary work provides new insights into oyster metabolism, and demonstrates the potential to pursue more complex metabolomic assessments of marine life using CSI techniques.

We have demonstrated that less than 10 minutes of scan time can generate excellent anatomic images of oysters and marine sponges in Figure 1 and 2, respectively. MR images between 0.1 to 0.2 mm pixel resolutions can be easily obtained. Along with the routine anatomic images, the first example of *in vivo* glycine metabolism in an oyster was demonstrated using ^13^C CSI, emphasizing the functional aspects of the data used to generate images.

Unlike the application of MRI in human clinical settings, MR research on marine animals requires hardware modification to accommodate different sized samples and unique life support systems. In addition to the range of sample sizes, the ionic components in sea water pose unique challenges for coils used for MR signal detection. The increased effective resistance due to the salinity of sea water and high ion contents of living samples decreases the quality factor (*Q*) of NMR RF coils and causes frequency shifts that must be adjusted by tuning and matching capacitors. The lower Q factor from the increased salinity requires the use of a larger and longer RF-pulse length. As other investigators have shown before [12,30,31], we had to adjust the capacity of the match and tune capacitors in the current NMR coil circuit extensively to minimize the negative effects of highly conductive media. Even with coil modifications, the much lower Q factor with saline loaded samples inside the RF coil will inevitably require much more RF power. Spin echo sequence is an excellent tool providing different contrast images with minor adjustment of timing between RF pulses and repetition time. Most of the MR images from marine samples reported so far have used simple spin echo sequences, which require exact 90 and 180 degree pulses to work most efficiently. The high energy RF pulses of spin echo sequences deposit RF energy sufficient to cause heating of the sample and this RF energy deposition is proportional to the square of the flip angle employed. More experimentation on the judicious choice of pulse sequences that can generate the intended contrast without using the high flip angle and RF power of spin echo techniques can be justified for the future application of marine MRI. For example, variation of turbo gradient spin echo methods such as GRASE (gradient echo and spin echo)[32], acquires more echoes than spin echo sequences, and can be used to acquire images faster or with higher resolution. Also, customizing the pulse sequence based on the gradient echo techniques including the use of variations such as FLASH (fast low angle shot) and SSFP (steady state free precession) for ultra fast imaging experiments [33,34] can change the image contrasts through adjusting parameters such as repetition time, echo time and flip angle. Using a modified spin echo sequence with a reduced refocussing RF pulse angle in spin echo, will reduce the required RF power significantly compared to conventional spin echo sequences. With these sequences, image contrast can be easily controlled by putting a magnetization preparation step before the start of the sequence. For example, these sequences are more suitable for obtaining an image with contrasts depending on perfusion, diffusion and flow. Even though MR images of excellent planar resolution have been obtained using multi-slice spin echo or a variation of it, typical resolutions along the slice direction are orders of magnitude larger as shown in our examples and those of others. The true potential of MR application to marine science probably lies with the three dimensional imaging techniques. MR is intrinsically adapted to volumetric data and the reformatting the data to display any cross-sectional detail is a routine operation [34]. In our laboratory, three dimensional MR imaging techniques are most often used to acquire images from small vertebrates including sea turtles to limit the size of data sets required to handle the needed resolution of between 70 to 120 microns isotropically. From our experience, variations of gradient echo techniques (FLASH and SSFP) turned out to be more flexible for various 3-D acquisitions than spin echo sequences.

The MR spectroscopy techniques can be grouped into either single voxel or multi voxel methods. Single voxel techniques have advantages such as the volume defined is highly selective with minimal contamination from the surrounding tissue and there is excellent spectral resolution due to better shimming which is more easily achieved. The results from an experiment can be quickly examined after simple signal processing. On the negative side, the whole procedure has to be repeated to obtain the data from other parts of regions of interest. In this respect, the multi voxel techniques such as CSI can be thought of as complementary to the single voxel techniques. The CSI techniques can provide spectra from many different areas simultaneously, but they are more susceptible to spectral distortion due to magnetic field inhomogeneity and intra-voxel contamination. Processing and display require several operator inputs making unbiased examination of the data more challenging. The effects of most commonly applied processing steps, such as eddy current correction with a water reference, baseline correction, spatial apodization, zero filling, voxel shifting, and main magnetic field shift correction, are well enough understood to be mentioned without much further comment [20]. In ^13^C CSI experiments, unlike to ^1^H CSI experiments, water suppression and base line correction to remove the rolling baseline from fat and macromolecules can be omitted.

The main drawback of the CSI techniques, especially when dealing with nuclei other than protons, is the long acquisition time required to increase the signal to noise ratio in the final processed CSI data, and the additional phase encoding steps to resolve the spatial information. Reduction of experimental time in CSI is currently an active area of research and techniques based on echo planar imaging (EPSI, echo planar spectroscopic imaging), parallel imaging and hyper-polarized molecules are available and need to be explored [28,35,36].

It will be a potential concern in future high resolution MR studies of marine organisms that motion degrades image quality so severely as to obscure details of the structure as shown in Figure 2. To reduce the motion artifacts, either faster acquisition or gating techniques synchronizing the data acquisition to the motion of the subject are usually applied in clinical applications. Fast image acquisition techniques can both reduce the motion artifacts and capture the physiological changes caused by experimental manipulations. When appropriate environmental conditions are maintained, the duration of experimental time is not necessarily a limiting factor in marine samples and we can repeat data acquisition many times to accumulate excellent SNR if the motion during each period can be controlled. Effects of motion on image quality can also be reduced using gating to synchronize the repetition of the imaging sequence with periodic motion of the subject. Measurement of a physical property can also be used to provide a trigger to synchronize the imaging sequence to motion, so that the subject appears to be static in terms of the MR data acquisition process. Electrical signals generated during the motion of the object or the pulsatile flow cycle of the sea water in the life support chamber can be used. This signal is conducted to the console and triggers the pulses for the start of the imaging sequence.

## 3. Experimental Section

The CSI technique used was a modification of an ordinary imaging technique but adds a chemical shift as an additional dimension to obtain the necessary spatial information. In the basic imaging technique, one spatial direction is resolved during data acquisition by a gradient (read-out gradient) which modulates the frequencies according to the coordinates along the applied gradient direction. In the CSI technique, during data acquisition period, this gradient is omitted to preserve the chemical shift information and this additional dimension creates a 2-dimensional or 3-dimensional data array from the 1- or 2-dimensional object image. Because of this additional spectral dimension, the acquisition time will often be unacceptably long, especially when three- and four-dimensional CSI experiments are required, because the phase encoding gradients along one spatial direction should be applied independently from each direction. To shorten experimental time, either the spatial resolution and/or the spatial dimensions are reduced, or fast acquisition techniques are developed [20,35,37]. Another approach is to skip the phase encoding steps located in the corners of k-space, sampling only k-space with low frequencies. The missing data points are filled with zeros and the resulting data file looks like a regular rectangular data matrix suitable for routine transformation with a lower spatial resolution. To maximize the signal to noise ratio (SNR) for a given experimental time, a variable number of averages for each phase encoding step are performed depending on the position in k-space, typically predetermined by a Hanning shaped weighting function. Compared to MRI examination, the MR spectroscopy methods based on either CSI or single voxel techniques are more demanding on magnet homogeneity and spatial selection to reduce artifacts from broadened line-width and spectral contamination from larger water and lipid peaks which can overwhelm much smaller metabolite signals.

The typical preprocessing steps of CSI data we used included k-space filtering, zero filling, apodization along the spectral dimension, and Fourier transformation. Before using the automatic peak integration routine, to separate macromolecules with broader resonances from the narrower metabolite peaks, the spectra were subjected to removal of baseline roll. This removes very low frequency components due to unsuppressed water and 1st order phase changes from acquisition timing errors and frequency-shifts caused by magnet instability and phase corrections. Peak heights were estimated using a priori knowledge about the relative positions of the major peaks for quantification. The results of the CSI experiments were displayed as either a small array of spectra aligned according to their spatial origins or so-called metabolic maps which are false color intensity maps created using the peak integration values of predefined spectral ranges of observed metabolites which are overlaid over the reference proton image. More complicated CSI specific post-processing options necessary to choose spectral and spatial data and, especially, the need for a common platform to display the huge spectral and spatial information for easy visualization are the main reasons for slow adaptation of this versatile technique outside the research community [36,38].

### MR acquisition

MR imaging and spectroscopy were performed using a Varian 4.7T INOVA MRI system (Varian, Inc, Palo Alto, CA, USA) with a 33 cm horizontal bore magnet equipped with 20cm inside diameter gradient coil insert with integrated shims (Model BFG-300/200, Resonance Research Inc, Billerica, MA, USA). The maximum gradient strength was 300 mT/m. At this field strength, the resonant frequencies of proton and carbon were 200 and 50 MHz, respectively. To demonstrate typical images using this hardware setup, *in vivo* MR images of oyster and sponge were chosen. Different contrasts, depending on the timing of the pulse sequence, can be obtained.

A phantom was constructed to test the ability of our protocols to acquire proton CSI data. The phantom consisted of a 50 mL polypropylene conical tube containing two 0.5 mL centrifuge tubes filled with corn oil and then itself filled with 30 mM isopropyl alcohol. A 72 mm diameter volume coil was used as a transmitter and a 2 cm diameter surface coil, placed directly over the phantom was used as a receiver. After acquiring 3-plane localizer images using a gradient echo sequence (repetition time (TR)/echo time (TE) = 100 ms/10 ms, 64 × 64 pixel resolution, 1 mm slice thickness), global shimming was performed by applying the automated shimming routine FASTMAP [39] to the region of interest encompassing the sample. After obtaining 3-plane localizer images, reference images were acquired using a rapid acquisition with a relaxation enhance (RARE) sequence [40] using the following parameters: TR/TE = 1,500 ms/8 ms, four echo trains per each TR period, field of view = 5 cm × 5 cm, matrix size = 256 × 256 and slice thickness = 1 mm. The CSI sequence consisted of a double spin echo sequence and 16 × 16 phase encodings using 2 mm slice thickness. The TR/TE was 1000 ms/15 ms. Chemical shift selective pulses with dephasing gradients were applied for water suppression. The field of view was 50 × 50 mm and the volume of interest was 18.6 × 10 × 2 mm selected using a point resolved spectroscopy method [25]. Six additional outer volume suppression pulses were graphically assigned over the reference image and applied before the start of the CSI sequence, resulting in six outer volume suppression slabs of 1-cm thickness each on all sides of the volume of interest. After the end of the data acquisition, the CSI data was extrapolated to 32 × 32 to yield a nominal voxel size of 1.56 × 1.56 × 2 mm.

An oyster was injected with ^13^C enriched glycine. Twelve hours following injection, ^13^C CSI experiments were performed as follows: two coils with different resonance frequencies are needed to simultaneously transmit and acquire signals from proton (200 MHz) and carbon (50 MHz). The coil assembly for the ^13^C CSI experiment on the oyster consisted of an 8 cm diameter proton volume coil and a 2.5 cm diameter carbon linearly polarized surface coil. The proton coil was used for both imaging and decoupling purposes [41]. The RF pulse power of the ^13^C coil was calibrated in a separate phantom experiment to produce a 90 degree pulse at the depth of 5 mm from the surface of the coil. To reduce interference between the proton and carbon channels, a high pass filter (Trilithic, Indianapolis, IN, USA) was inserted into the line from the proton coil and a lowpass filter was inserted into the carbon channel line between the RF coil and the T/R switch. First, proton images were acquired along the 3 different axes and these proton images were used to confirm the oyster position in the magnet and overlay the ^13^C CSI spectra acquired later for better visualization of spatial origins of ^13^C spectra, similar to ^1^H CSI experiment shown in Figure 3. A rapid acquisition using a relaxation enhance (RARE) sequence was used for this. An automated shimming routine FASTMAP was applied to the region of interest. Two dimensional (2-D) ^1^H-decoupled ^13^C MRSI sequences using a non-selective excitation RF pulse were used to acquire two spatial and one spectral dimension of data. Coupling between proton and carbon nuclei in the molecule leads to splitting of peaks and therefore reduces the SNR by spreading the intensity of the peaks into the multiples. This complicates the analysis of the results due to overlapping of peaks. This can be avoided by collapsing the multiplet to a single peak by decoupling. A proton decoupling scheme using WALTZ-16 was on only during the data acquisition period of 0.128 sec to minimize possible heating of the oyster [42]. The 2-D spatial dimensions were parallel to the plane of the ^13^C surface coil to minimize the effect of RF inhomogeneity of the surface coil. The nominal matrix size along the spatial dimensions was 16 × 16 with a Hanning-weighted k-space sampling scheme [9]. The spectral bandwidth was 5 KHz and 512 spectral data points. TR was 1 sec and the total acquisition time was about 2 h. After data collection, the k-space matrix was zerofilled to 128 × 128 along the spatial dimensions and 10 Hz Lorentzian line broadening applied along the spectral dimension. Fourier transformations of spatial and spectral dimensions were done to generate a 3D data set. The metabolite maps were generated according to the area under the curve after fitting the spectral component with Lorentzian line shape and zero-filling to 128 × 128, similar to ^1^H CSI experiment shown in Figure 3.

## 4. Conclusions

In summary, applications of MRI, MRS, and CSI to marine sciences are rare compared to the human clinical realm. There are hardware and software issues associated with the high ionic strength of the seawater that were demonstrated to not be a serious problem for metabolic imaging. As environmental metabolomics becomes more accepted in the environmental sciences and with more access to MRI instrumentation, novel discoveries in marine sciences will rapidly follow. The non*Mar.* invasive, dynamic inherent aspects of MRI and MRS permit longitudinal studies to be performed to elucidate long-term metabolic adaptations and permit the correlation of the genome to downstream effects on the metabolome. Specifically, metabolomic imaging has the potential to distinguish sublethal effects of perturbations of the metabolism of marine organisms, including those impacts that may be due to changes to the ocean environment. Combined ^13^C and ^1^H CSI will be a powerful technique to determine whole distribution kinetics and dynamics of ^13^C-labeled substrates.
